# Considerations Regarding Online Group Psychotherapy Sessions for Breast Cancer Patients in Active Phase of Oncological Treatment

**DOI:** 10.3390/healthcare11162311

**Published:** 2023-08-16

**Authors:** Elena Gabriela Vâlcu, Dorel Firescu, Aurel Nechita, Anamaria Ciubară, Georgiana Bianca Constantin, Gabriela Rahnea-Nita, Laura-Florentina Rebegea

**Affiliations:** 1 School of Advanced Doctoral Studies, “Dunarea de Jos” University, 800008 Galati, Romania; elenag.valcu@gmail.com (E.G.V.); dorel.firescu@ugal.ro (D.F.); aurel.nechita@ugal.ro (A.N.); anamaria.ciubara@ugal.ro (A.C.); laura.rebegea@ugal.ro (L.-F.R.); 2 Faculty of Medicine and Pharmacy, “Dunarea de Jos” University, 800008 Galati, Romania; 3 Department of Pediatrics, “Sf. Ioan” Clinical Hospital for Children, 800487 Galati, Romania; 4 Clinical Department, Faculty of Medicine, Carol Davila University of Medicine and Pharmacy, 050474 Bucharest, Romania; gabriela_rahnea@yahoo.com; 5 “Sf. Luca” Chronic Diseases Hospital, 041915 Bucharest, Romania; 6 Department of Oncology, “Sf. Andrei” County Emergency Clinical Hospital, 800179 Galati, Romania

**Keywords:** online group therapy, cyberspace, breast cancer, EQ-5D-5L (European Quality of Life), depression, anxiety

## Abstract

Introduction: The aim of this study is to evaluate the results of online group meetings for breast cancer patients in the active phase of treatment. The group therapy sessions took place weekly, synchronously, online, on Zoom, with a total of 12 meetings lasting about 2.5 h per session, between December 2021 and February 2022. We analyzed the topics of discussion chosen by the participants, the structure of the group, the results obtained at the main scales of evaluation/monitoring of quality of life and the motivation of patients to participate in the therapeutic group. All patients were in the active phase of treatment (chemotherapy, radiotherapy, hormone therapy, etc.). The main goal of the group therapy was to reduce the stress related to the disease. Material and methods: Systematic observations included ABS psychological tests, EQ-5D-5L, HADS—Hospital Anxiety and Depression Scale, and the Recurrence Fear Questionnaire; the questionnaires were administered at the beginning and at the end of the therapeutic intervention; the participation in the therapy and in the research of the patients began after signing the informed consent document; the intervention was evaluated at the end using a feedback questionnaire. The group was closed, and the participants signed an informed consent document and agreed to have the sessions recorded. Results: Comparing the initial with the final results of the psychological tests administered, there was an improvement in the quality of life of the participants in all areas, with a clinically significant decrease in the areas of pain and depression, along with an increase in perception of well-being, a decrease in FoP scores and an increase in the level of rationality about the disease. Conclusions: Group therapy for cancer patients was useful in improving the quality of life; the closed group, even online, provided a safe environment in which they could share feelings. A close correlation was noted between the scores obtained on the FoP-Q and HADS scales. It is evident that there is a strong relationship between FoP and depression. Results on these scales correlated well with results on the EQ-5D-5L quality of life questionnaire.

## 1. Introduction

**Figure 1:**
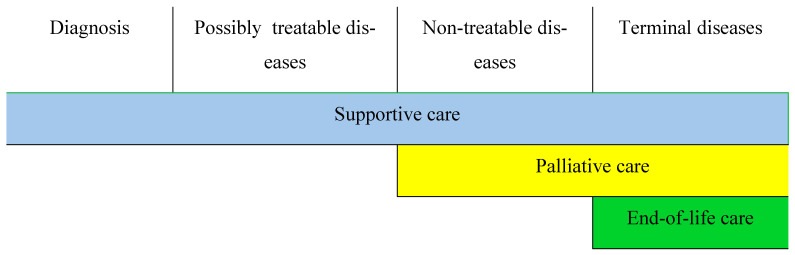
The relationship between supportive, palliative and end-of-life care (EoL) depending on the evolution of the disease [2].

The World Health Organization (WHO) explains palliative care as an approach that improves the quality of life of both patients and their families, while dealing with the problems associated with chronic incurable diseases, by preventing and alleviating suffering, early identifying, evaluating and treating pain flawlessly, as well as mitigating other physical, psychosocial and spiritual problems [1] (Figure 1).

Supportive, palliative and end-of-life care (EoL care) are related concepts [1].

Breast cancer is the most common cause of cancer morbidity and mortality in women worldwide [1], even though it is largely preventable through population screening. Despite notable progress in recent years in the EU, breast cancer screening programs still maintain large variations between countries and socio-economic groups. In general, in recent years, medicine has firmly stated that its ultimate goal is to respond to the legitimate need for a satisfactory quality of life [2] and in achieving this goal, medicine seems benevolent in receiving psychological themes and suggestions. Quality of life (QoL) represents, in this sense, a concept that integrates psychological, social, economic and functional aspects.

Romania is facing an increase in the number of neoplastic patients, as it does not have a prepared health system [3].

The level of life expectancy in Romania (a significant indicator of the quality of life) increased by 1.8 years, according to Eurostat data, remaining among the lowest in the EU and almost six years below the EU average.

Psychotherapeutic intervention in the context of oncological treatment involves overcoming the somatic–psychic dichotomy and taking into account the complex dynamics of this relationship [4,5]. Over time, this idea has appeared/re-emerged cyclically and recent neuropsychology and psychoneuroimmunology studies [5] demonstrate that psychological factors can affect the body’s immune function and therefore its response to diseases. In the pandemic context, due to the restrictions imposed by the authorities, the Internet has made therapy accessible. Moreover, the Internet made therapy accessible to patients who could not travel or lived far away from a psychological office.

In another study [6], the authors proposed a model for measuring the effectiveness of psychological interventions that produce sequential results in different domains, each one preparatory for the next one:

1. Subjective well-being, sense of mastery, hope (remoralization);
2. Reducing symptoms, activating healthy coping strategies and solving life problems;
3. Social functioning, lifestyle, achievement of new goals (rehabilitation).

These general considerations were later reshaped, taking into account the oncological field of application which is often characterized by a short intervention time and foresees psychological and/or spiritual suffering conditioned by the existential condition of a disease with a dire prognosis, and also taking into account the pandemic context and how group therapy can become more effective [7].

Mihoc, Pustianu and Degi (2021) showed in the study entitled “Psycho-oncology in Romania. New perspectives and research directions” that among Romanian oncological patients, almost half of them present depressive or anxious symptoms. The diagnosis of cancer is a traumatic event, involving physical symptoms (fatigue, nausea, pain), symptoms of anxiety or depression, social stigmatization, spiritual problems (loss of meaning in life), and financial problems that complicate the survival process and considerably influence the patient’s quality of life [8]. The adaptation to the oncological diagnosis is a complex process that begins with the identification and recognition of the oncological distress [8,9,10].

The present study followed the processes carried out in the virtual space and highlighted the mechanisms that work in online therapy, as well as the responsibilities of the group leader, ending with a pre- and post-therapy analysis of the results obtained in the administered psychological tests.

## 2. Materials and Methods

### 2.1. Individual Pre-Group Psychological Assessment

Before forming the group, the patients participated in individual online meetings with the therapist. The purpose of these preliminary meetings was to evaluate the personality of each individual and implicitly to create the therapeutic alliance. As a methodology, a semi-structured questionnaire was administered [11] with the 7 steps of an effective interview [12,13]:

1. Empathy.
2. Defining the problem/list of problems.
3. The therapeutic contract/therapeutic goals pursued (examples reported by the participants: reducing anxiety, increasing communication skills, reducing the state of physical, mental and emotional exhaustion, forming strategies to accept the disease and fight against it, etc.).
4. Identification of dysfunctional processes.
5. Strategies.
6. Technics.
7. Verification.

### 2.2. Psychological Evaluation-Group Characteristics

The psychological tests used in the evaluation of the group were the EQ-5D-5L Questionnaire (to measure the quality of life in breast cancer patients), HADS—(scale that measures the degree of anxiety and depression), Fear of Recurrence Questionnaire (FoP-Q -SF). Using these samples, the specific symptoms of psychopathology, personality factors, and behavioral reactions in stressful situations were evaluated.

Regarding the results obtained by the participants in the EQ-5D-5L Questionnaire, they did not report any mobility problems. Most reported problems were in the area of pain and discomfort, as well as difficulties in usual activities. Five of the participants indicated a medium level of depression and four of them indicated a high level of anxiety (three of the participants presented both depressive and anxious symptoms). These latter results correlated with those obtained using the HADS scale. High anxiety scores were also obtained using the FoP-Q scale. At the beginning of the therapy, the patients did not register a high level of depression on this scale, but they did register a high level of anxiety. Four patients obtained high values (over 37) on the FoP-Q scale. The results of the ABS questionnaire (which assesses irrational beliefs about illness) highlighted the positive association between irrational beliefs and emotional distress, as well as the positive association between irrational beliefs and anxiety and depression.

Therefore, we can say that the patients who form the group are characterized by anxious–depressive tendencies supported by introjected aggressive aspects, such as self-blame, the conviction that they must suffer because of their sins, which makes them neglect their own needs and leads to a weak self-awareness which does not allow adequate self-definition. Based on these beliefs, patients tend to structure their interpersonal relationships against the background of affective anesthesia. Mistrust and negative expectations tend to prevail in their interpretation of reality. They are predominantly anxious people, who control their own state of tension incongruously. The aspects of hopelessness, helplessness, experiences of abandonment, traits of inhibition and inertia, and poor ability to discharge tension through action confirm the description of Temoshok-Morris-Greer-Grossarth-Maticeck (2009) [14], defined as a “type C of personality pattern” (personality predisposed to cancer) in which the suppression of emotional responses (anger), conformity, lack of assertiveness and an “expectation of external control” predominate [15]. The patients examined demonstrate a lack of self-perception and a lack of assertiveness and, therefore, show low resistance to stress. Also, based on the described characteristics, we can say that the examined patients could have maladaptive coping styles. Such a hypothesis in turn aligns with the position of other authors [16], who claims that people affected by neoplastic diseases (determined by external stressors related to separation–loss events) have a tendency towards passivity and giving up.

## 3. Research Design

The therapy group consists of nine patients diagnosed with breast cancer in the active phase of treatment, between 32 and 55 years old, middle–higher education, married, with minor and adult children, living in different cities of Romania: Bucharest, Bacău, Ialomița, Constanța, Galaţi, Timişoara, and Odorheiu Secuiesc.

Methods design: Systematic observations, psychological tests ABS-2 [17], EQ-5D-5L [18,19], HADS [19]–Hospital Anxiety and Depression Scale, FoP-Q [20] Fear of Recidivism Questionnaire; the samples were administered at the beginning and at the end of the therapeutic intervention in order to verify the effectiveness of the online group therapy. The intervention was evaluated at the end by including a Feedback Questionnaire. The group therapy sessions were held online synchronously, on Zoom, with weekly frequency, comprising 12 meetings with a duration of about 2.5 h per session, between December 2021 and February 2022. In the online group therapy, individual pre-group meetings (on the Internet) were established with each participant (Table 1).

The group was closed; the participants signed an informed consent document and accepted that the sessions were recorded. The size of the group wanted to be narrowed down to an entity character. It is known that with the increase in the number of members, the tendency to fractionalize, to form subgroups, often increases. On the contrary, a small number facilitates the formation of emotional bonds. All patients were in the active phase of treatment (chemotherapy, radiotherapy, hormonal therapy, etc.). The discussion topics during the sessions were chosen by the participants based on the majority criteria.

The main goal of group therapy was to reduce oncological distress. In creating the group, the criteria of homogeneity (same type of cancer, similar stage of the disease) and heterogeneity in terms of socio-demographic characteristics (age, educational level, profession, etc.) and interpersonal difficulties were taken into account.

The initial psychodiagnostic evaluation therefore confirmed the hypothesis (also present in the specialized literature) of the existence, in patients with breast cancer, of dysthymic aspects in the premorbid personality, which, in pathological situations, seem to be organized as an adjustment disorder with Depressive Mood (DSM-5) [21,22]. This is the clinical picture that was observed after the diagnosis and at the time of the proposed intervention. From the beginning, it was possible to observe how the patients experienced the psychotherapeutic moment as a place available to them where they can express themselves without fear of being judged. In this context, therefore, they were able to refer to many aspects, such as deprivation and inadequacy related to self-image and loss of femininity.

Entering the group psychotherapeutic framework has an effect of temporal suspension “here and now”, meaning entering a dimension of the psychological present. The group is more than the sum of its members. It (co)creates itself as an entity with a specific psychology.

### Group Interventional Program

**Figure 2:**
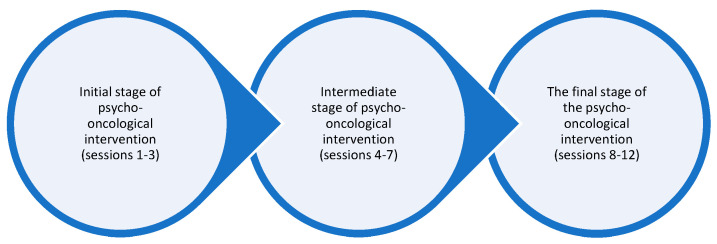
Stages of psycho-oncological intervention.

As a working methodology within the group, cognitive–behavioral interventions were applied (identifying irrational beliefs/thinking errors, automatic thoughts, generalizations, and finding alternative thoughts); interventions from transactional analysis: life scenarios, injunctions, etc.; the Simonton method: readings/relaxation exercises, stressor analysis, physical exercises, imagery of relapse and death, and inner guidance. The group intervention program has a staged structure and consists of 12 therapy sessions as seen in Figure 2:

The initial stage of psycho-oncological intervention

Objectives:

- Building a therapeutic relationship characterized by empathy, unconditional acceptance and congruence.
- Creating an overview of the main psychosocial needs.
- Normalization of emotional reactions.
- Reinforcement of already existing positive coping mechanisms.
- Creating new coping mechanisms.
- Providing emotional support.
- Providing informational support.

Activities:

- Individual pre-group interviews.
- Group rules.
- Presentation of informed consent.
- Presentations and expectations from group meetings.
- Psychoeducation.
- Sociometry.
- Administration of psychological tests.

Intermediate stage of psycho-oncological intervention

Objectives:

- Setting the main goals for a better adjustment to the neoplastic disease.
- Continue to provide emotional support.
- Introduction, conceptualization, and practice of validated techniques for oncological patients.

Activities:

- Choice of discussion topics based on majority criteria.
- Cognitive–behavioral psychological intervention, with cognitive restructuring techniques, problem-solving techniques, breathing techniques, relaxation, and guided imagery.

The final stage of psycho-oncological intervention

Objectives:

- Strengthening the mechanisms of adjustment to neoplastic disease.
- Preventing emotional relapses.
- Therapeutic approach to the fear of recurrence.
- Psychological reassessment, including scores of the emotional state.

Activities:

- Summarizing and practicing the information and skills learned during previous sessions.
- Magic Shop.
- Exit ritual.
- Re-administration of initial psychological tests and a feedback questionnaire.

It should be mentioned that it is very important to establish the therapeutic alliance from the beginning within the psychotherapeutic program, as well as to maintain it throughout the therapy.

## 4. Results and Discussions

**Figure 3:**
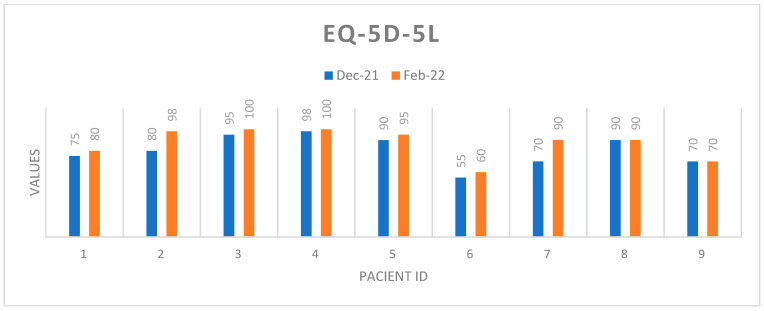
Self-assessment of health status.

At the end of the group therapy program, an increase in self-rated health was noted. Also, HADS scores showed a clinically significant decrease at the end of therapy on both the depression and anxiety dimensions. Three patients achieved clinically significant lower scores at the end of therapy, according to the FoP-Q-SF questionnaire. In correlation with the results obtained using the HADS Scale, in the same patients, a slight increase in the level of rationality about the disease was also noted (Figure 3). Rational beliefs have a protective role, leading to functional or adaptive negative emotions.

### Profiles EQ-5D-5L

**Figure 4:**
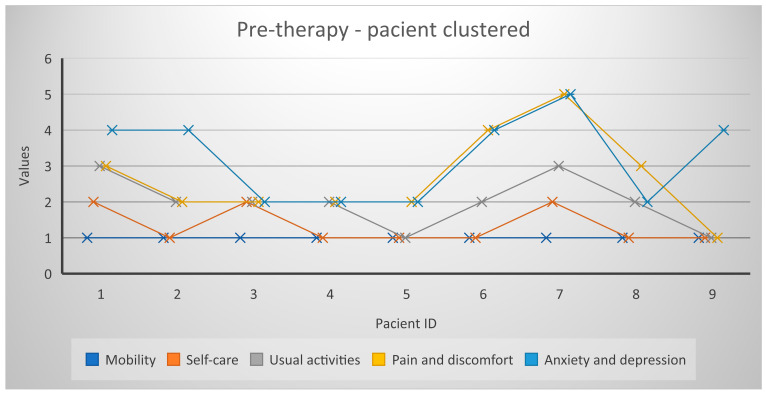
Profiles EQ-5D-5L/December 2021.

**Figure 5:**
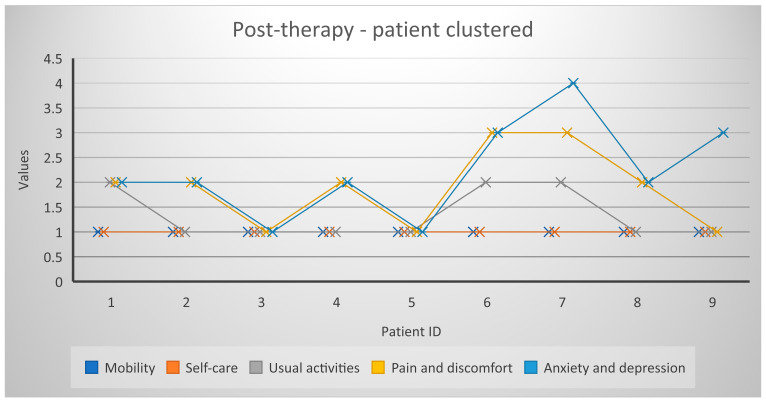
Profiles EQ-5D-5L/February 2022.

Figure 4 and Figure 5 represent a global picture of the results obtained in the quality of life questionnaire. It is noted that improvements were spread across all dimensions and EQ-5D-5L scores decreased significantly at the end of therapy.

The conversion of EQ-5D profiles into EQ-5D index scores allowed us to obtain the data below (Table 2), which is necessary for the activities of monitoring the quality of life of patients undergoing medical treatment. These data can range from 0 to 1 (perfect health).

**Figure 6:**
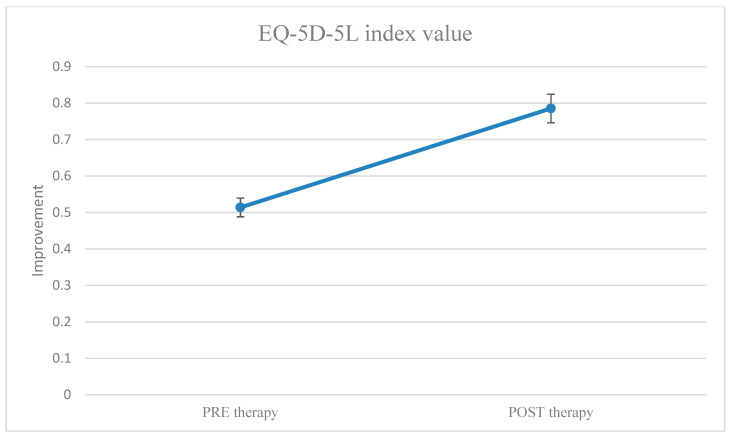
EQ-5D index score values before and after group therapy. EQ-5D index anchored between 0 and 1 (1 = perfect health).

Figure 6 shows that the group therapy participants perceived an improvement in their quality of life.

Given that nine individuals participated in the group therapy (a small number from a statistical point of view, but important to ensure the group unity), to support the fact that there is a statistical significance, we used the MedCalc software.

The independent samples t-test was used to test the hypothesis that the difference between the means of two samples is equal to zero. The program displays the difference between the two means, and the 95% Confidence Interval (CI) of this difference. When the *p*-value is less than the conventional 0.05, the null hypothesis is rejected and the conclusion is that the two means do indeed differ significantly.

To establish the threshold (which indicates that the hypothesis is valid H0 or H1: H0—there is no difference between the means of the parameters before and after the group therapy, meaning the therapy has no effect, H1—there is a significant difference, so the therapy has an effect; the threshold value is 0.05), we used the T significance test, performing the following steps:

- We checked if the paired variables (before–after therapy) have a normal distribution (Statistics—Summary statistics)):

VariableEQ_5D_5L_index_value EQ-5D-5L index valueSample size9Lowest value0.09500Highest value0.7680Arithmetic mean0.534995% CI for the mean0.3610 to 0.7087Median0.635095% CI for the median0.3321 to 0.7326Variance0.05116Standard deviation0.2262Relative standard deviation0.4228 (42.28%)Standard error of the mean0.07539Coefficient of Skewness−0.9585 (*p* = 0.1766)Coefficient of Kurtosis0.1095 (*p* = 0.7872)D’Agostino–Pearson test for Normal distributionaccept Normality (*p* = 0.3869)Percentiles
95% Confidence interval250.3725
95

VariableEQ_5D_5L_index_value_1 EQ-5D-5L index value_1Sample size9Lowest value0.4830Highest value1.0000Arithmetic mean0.785295% CI for the mean0.6640 to 0.9064Median0.768095% CI for the median0.7015 to 0.9790Variance0.02485Standard deviation0.1576Relative standard deviation0.2008 (20.08%)Standard error of the mean0.05255Coefficient of Skewness−0.3440 (*p* = 0.6226)Coefficient of Kurtosis0.9296 (*p* = 0.4178)D’Agostino–Pearson test for Normal distributionaccept Normality (*p* = 0.6380)Percentiles
95% Confidence interval250.7260
95

- It became evident that both have a normal distribution, so we applied the T-test (Statistics-T-tests-Independent Samples).
- The result was *p* = 0.015 (*p* < 0.05), so the hypothesis H0 is false and there are significant differences between the values of the variable EQ-5D-5L index value (before therapy) and EQ-5D-5L index value_1 (after therapy), meaning that the group therapy had an effect.

Sample 1VariableEQ_5D_5L_index_value EQ-5D-5L index valueSample 2VariableEQ_5D_5L_index_value_1 EQ-5D-5L index value_1
Sample 1Sample 2Sample size99Arithmetic mean0.53490.785295% CI for the mean0.3610 to 0.70870.6640 to 0.9064Variance0.051160.02485Standard deviation0.22620.1576Standard error of the mean0.075390.05255F-test for equal variances*p* = 0.327

T-test (assuming equal variances)
Difference0.2503Standard Error0.0919095% CI of difference0.05552 to 0.4451Test statistic t2.724Degrees of Freedom (DF)16Two-tailed probability*p* = 0.0150

One year after the completion of the group therapy, a group follow-up session was held online, lasting about 3 h, during which the participants demonstrated active behaviors, asking each other questions related to the disease, personal, professional life, etc., in a positive affective climate. The participants reported that they continued to visit each other (those who lived nearby), spent time together, and supported each other in difficult moments (finding out medical results, before surgical interventions, etc.). Specifically, one patient exposed her “new breasts” (after breast reconstruction), encouraging the others to do as well, stating: “it’s worth the effort, now I feel more feminine, more beautiful, I notice the admiring glances around me.” Most of the patients resumed their professional activity, declaring themselves very satisfied, while others changed their occupation. Two of the participants congratulated themselves and were congratulated by the group for ending dysfunctional romantic relationships (which began before the cancer diagnosis). Two other participants reported no significant changes in quality of life since the end of therapy. One patient reported a worsening of her health. Moreover, the participants expressed their gratitude towards the existence of the group. It should be specified that the group still exists in an asynchronous online format on WhatsApp.

**Figure 7:**
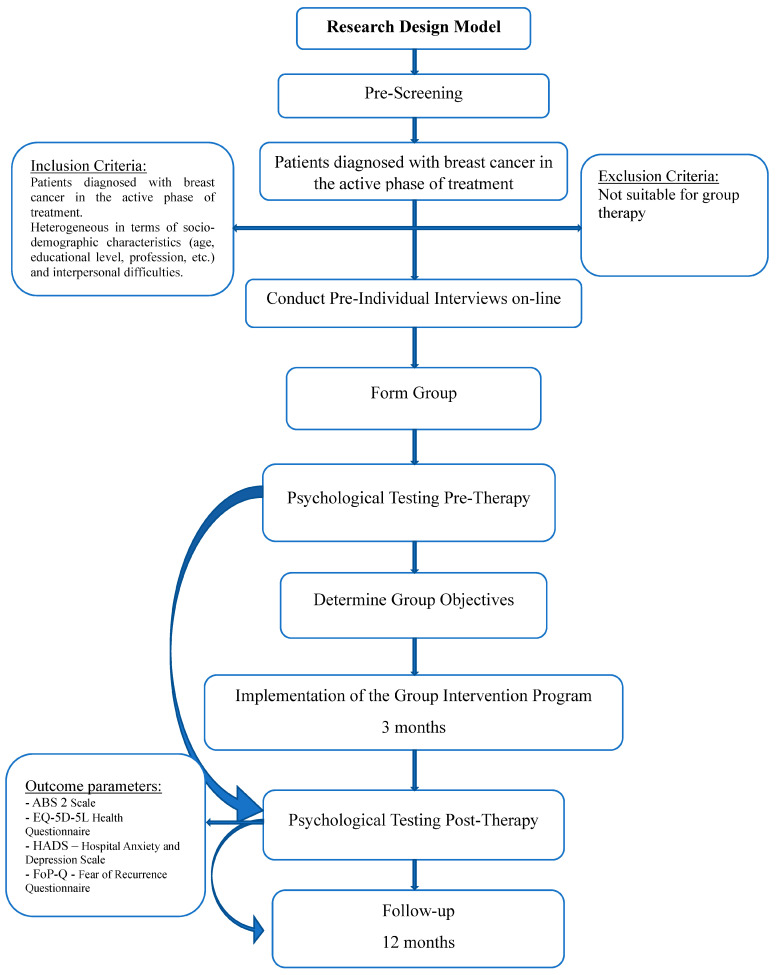
Flowchart of the study.

To synthesize, Figure 7 describes a summary of the study:

## 5. Conclusions

A close correlation was noted between the scores obtained on the FoP-Q and HADS scales. It is evident that there is a strong relationship between FoP and depression. Results on these scales correlated with results of the EQ-5D-5L quality of life questionnaire. The FoP-Q is more closely related to the clinical side and has been well accepted by patients because it is short, concise, and easy to understand.

Group therapy for cancer patients was helpful for improving quality of life, as the closed group, even online, provided a safe environment in which they could express themselves. We believe that the inoculation of hope, catharsis, transmission of information, and altruism, as therapeutic factors were transmitted within the online psychotherapy group. Relationships based on trust, acceptance, and confidence were established. The participants felt the need to share their pain, trauma, animosities in relationships with medical personnel, experiences in their romantic relationships, and the relationship with their own person. The goals of the meetings to activate the desire to fight, to live, to take responsibility, to find resources and to access them were achieved within the group therapy.

At the end of the therapy, the patients declared themselves stronger in the fight against cancer, more confident and better informed. The innovation of this study is the evaluation of an online therapy group for breast cancer patients, which, to the best of our knowledge, is the first study of this type published in Romania.

The novelty of this study consists of conducting a group therapy that started on the Internet in synchronous mode, then in an asynchronous mode and which led to the creation of friendships that translated into face-to-face relationships. Moreover, this study allowed us to highlight the fact that psychotherapeutic interventions used in face-to-face groups find their applicability in the online environment as well.

Limitations of the study: We have designed the study for a particular sex and disease, so it could be useful to have some other similar study with which to compare the results; the Hawthorne effect: participants may influence the data collected by changing their behavior when they know they are being observed; studies including more therapy groups may be required in order to validate the results of this study.

## Figures and Tables

**Table 1:** Structure of the therapy group.

Structure of the Therapy Group	
Number of sessions	12
Session duration	2.5 h
Materials presented	Informative materials about anxiety, oncologic distress, coping methods, relaxation techniques, etc.
Psychological instruments	- Semistructured interview (24 questions) - ABS 2 scale - EQ-5D-5L health questionnaire - HADS—Hospital Anxiety and Depression Scale - Fear of Recurrence Questionnaire (Fop-Q)

**Table 2:** EQ-5D scores PRE therapy and POST therapy.

	Mobility		Self-Care		Usual Activities		Pain and Discomfort		Anxiety and Depression	
Level	Pre-Therapy	Post-Therapy	Pre-Therapy	Post-Therapy	Pre-Therapy	Post-Therapy	Pre-Therapy	Post-Therapy	Pre-Therapy	Post-Therapy
1	9 (100%)	9 (100%)	6 (66.67%)	9 (100%)	2 (22.22%)	6 (66.67%)	1 (11.11%)	3 (33.33%)	0 (0%)	2 (22.22%)
2	0 (0%)	0 (0%)	3 (33.33%)	0 (0%)	5 (55.56%)	3 (33.33%)	4 (44.44%)	4 (44.44%)	4 (44.44%)	4 (44.44%)
3	0 (0%)	0 (0%)	0 (0%)	0 (0%)	2 (22.22%)	0 (0%)	2 (22.22%)	2 (22.22%)	0 (0%)	2 (22.22%)
4	0 (0%)	0 (0%)	0 (0%)	0 (0%)	0 (0%)	0 (0%)	1 (11.11%)	0 (0%)	4 (44.44%)	1 (11.11%)
5	0 (0%)	0 (0%)	0 (0%)	0 (0%)	0 (0%)	0 (0%)	1 (11.11%)	0 (0%)	1 (11.11%)	0 (0%)
Total	9 (100%)	9 (100%)	9 (100%)	9 (100%)	9 (100%)	9 (100%)	9 (100%)	9 (100%)	9 (100%)	9 (100%)
Number reporting some problems	0 (0%)	0 (0%)	3 (33.33%)	0 (0%)	7 (77.78%)	3 (33.33%)	8 (88.89%)	6 (66.67%)	9 (100%)	7 (77.78%)
Change in numbers reporting problems	0		−3		−4		−2		−2	
% change in numbers reporting problems	0%		−33%		−50%		−41%		−45%	
Rank of dimensions in terms of % changes	0		1		2		4		4	

## Data Availability

The datasets that support the findings in this article are not publicly available for privacy and security reasons but can be obtained from the corresponding authors upon reasonable request.

## References

[1] De Lima L. (2015). Palliative care and pain treatment in the global health agenda. Pain.

[2] Catane R., Cherny N., Kloke M., Tanneberger S., Schrijvers D. (2006). Handbook of Advanced Cancer Care.

[3] Furtunescu F., Bohiltea R.E., Voinea S., Georgescu T.A., Munteanu O., Neacsu A., Pop C.S. (2021). Breast cancer mortality gaps in Romanian women compared to the EU after 10 years of accession: Is breast cancer screening a priority for action in Romania? (Review of the Statistics). Exp. Ther. Med..

[4] Pai R., Nayak M., Sangeetha N. (2020). Palliative care challenges and strategies for the management amid COVID-19 pandemic in India: Perspectives of palliative care nurses, cancer patients and caregivers. Indian J. Palliat. Care.

[5] Mosoiu D., Mitrea N., Dumitrescu M. (2018). Palliative care in Romania. J. Pain Symptom Manag..

[6] Cardenas V., Rahman A., Zhu Y., Enguidanos S. (2022). Reluctance to Accept Palliative Care and Recommendations for Improvement: Findings from Semi-Structured Interviews with Patients and Caregivers. Am. J. Hosp. Palliat. Care.

[7] Jordan R., Allsop M., ElMokhallalati Y., Jackson C., Edwards H., Chapman E., Deliens L., Bennett M. (2020). Duration of palliative care before death in international routine practice: A systematic review and meta-analysis. BMC Med..

[8] Diaconu C., Maxim L., Timofte D., Livadariu L.M. (2014). Biopsychosocial Implications Related to the Breast Cancer in Young Women. Rev. Cercet. Interv. Soc..

[9] Ciuhu A.N., Rahnea-Nita G., Popescu M.T., Rahnea-Nita R.A. (2015). Evaluation of quality of life in patients with advanced and metastatic breast cancer proposed for palliative chemotherapy and best supportive care versus best supportive care. Cancer Res..

[10] Roca M., Mitu O., Roca I.C., Mitu F. (2015). Chronic Diseases—Medical and Social Aspects. Rev. Cercet. Interv. Soc..

[11] Schad F., Rieser T., Becker S., Groß J., Matthes H., Oei S.L., Thronicke A. (2023). Efficacy of Tango Argentino for Cancer-Associated Fatigue and Quality of Life in Breast Cancer Survivors: A Randomized Controlled Trial. Cancers.

[12] Rebegea L., Firescu D., Stoleriu G., Arbune M., Anghel R., Dumitru M., Mihailov R., Neagu A.I., Bacinschi X. (2022). Radiotherapy and immunotherapy, combined treatment for unresectable mucosal melanoma with vaginal origin. Appl. Sci..

[13] Gardikiotis I., Azoicai D., Dobreanu C., Petrescu I., Lazar A., Manole A., Ghetu N. (2016). Socio-Epidemiological Points of View Regarding Quality of Life in Patients with and without Breast Reconstruction after Mastectomy for Cancer. Rev. Cercet. Interv. Soc..

[14] Neagos A., Olteanu C., Manuc D., Bitere O.R., Cobzeanu M.D., Hinganu M.V., Cozma R.S. (2020). Quality of Life and Social Impact in Patiens with Laryngeal Tumors after Radiotherapy. Rev. Cercet. Interv. Soc..

[15] Aaronson N.K., Ahmedzai S., Bergman B., Bullinger M., Cull A., Duez N.J., Filiberti A., Flechtner H., Fleishman S.B., de Haes J.C.J.M. (1993). The European Organization for Research and Treatment of Cancer QLQ-C30: A Quality-of-Life Instrument for Use in International Clinical Trials in Oncology. J. Natl. Cancer Inst..

[16] Hansson E., Carlström E., Olsson L.-E., Nyman J., Koinberg I. (2017). Can a Person-Centred-Care Intervention Improve Health-Related Quality of Life in Patients with Head and Neck Cancer? A Randomized, Controlled Study. BMC Nurs..

[17] Monzio Compagnoni M., Caggiu G., Allevi L., Barbato A., Carle F., D’Avanzo B., Di Fiandra T., Ferrara L., Gaddini A., Giordani C. (2023). Assessment and Monitoring of the Quality of Clinical Pathways in Patients with Depressive Disorders: Results from a Multiregional Italian Investigation on Mental Health Care Quality (the QUADIM Project). J. Clin. Med..

[18] Mihoc A.R., Pustianu V.D., Degi L.C. (2021). Psycho-Oncology in Romania. New Perspectives and Research Directions. Rom. J. Med. Pract..

[19] Gaertner J., Siemens W., Meerpohl J.J., Antes G., Meffert C., Xander C., Stock S., Mueller D., Schwarzer G., Becker G. (2017). Effect of specialist palliative care services on quality of life in adults with advanced incurable illness in hospital, hospice, or community settings: Systematic review and meta-analysis. BMJ.

[20] Smith S.K., Loscalzo M., Mayer C., Rosenstein D.L. (2018). Best Practices in Oncology Distress Management: Beyond the Screen. Am. Soc. Clin. Oncol. Educ. Book.

[21] Kim S.J., Patel I., Park C., Shin D.I., Chang J. (2023). Palliative care and healthcare utilization among metastatic breast cancer patients in U.S. hospitals. Sci. Rep..

[22] Greer J.A., Moy B., El-Jawahri A., Jackson V.A., Kamdar M., Jacobsen J., Lindvall C., Shin J.A., Rinaldi S., Carlson H.A. (2022). Randomized Trial of a Palliative Care Intervention to Improve End-of-Life Care Discussions in Patients with Metastatic Breast Cancer. J. Natl. Compr. Cancer Netw..

